# One-Step Acidic Hydrothermal Preparation of Dendritic Rutile TiO_2_ Nanorods for Photocatalytic Performance

**DOI:** 10.3390/nano8090683

**Published:** 2018-09-01

**Authors:** Cheng Gong, Jun Du, Xiuyun Li, Zhenjie Yu, Jiansong Ma, Wenqian Qi, Kai Zhang, Jin Yang, Mei Luo, Hailong Peng

**Affiliations:** 1 Department of Chemical Engineering, School of Environmental and Chemical Engineering, Nanchang University, Nanchang 330031, China; stokescheng@sina.com (C.G.); show7898@foxmail.com (X.L.); topnevermiss@yahoo.com (Z.Y.); majiansong2330@sina.com (J.M.); qiwenqian9873@sina.com (W.Q.); zhangkai7755@sina.com (K.Z.); yangjin199501@sina.com (J.Y.); luomei@ncu.edu.cn (M.L.); 2 Key Lab of Poyang Lake Ecology and Bio-resource Utilization (Ministry of Education), Nanchang University, Nanchang 330031, China; 3 Jiangxi Province Key Laboratory of Edible and Medicinal Plant Resources, Nanchang University, Nanchang 330031, China

**Keywords:** titanium dioxide, nanorods, photocatalysis, acidic hydrothermal method, hydrophilicity

## Abstract

Three-dimensional and dendritic rutile TiO_2_ nanorods were successfully fabricated on a Ti foil surface using a one-step acidic hydrothermal method. The TiO_2_ nanorods were characterized using X-ray diffraction (XRD), energy dispersive X-ray spectrometry (EDX), transmission electron microscopy (TEM), scanning electron microscopy (SEM), and optical contact angle testing. The results showed that the nanorods with diameters of 100–500 nm and lengths of 100 nm to 1 μm were obtained on the Ti foil surface. The length and density of the TiO_2_ nanorods were perfect at the conditions of HCl concentration 0.5 mol/L, temperature 220 °C, and reaction time 12 h. The TiO_2_ nanorods formed parallel to the consumption of Ti and grew along the (110) direction having a tetragonal rutile crystal. The morphology of the nanorods possessed a three-dimensional structure. The contact angle of the nanorods was only 13 ± 3.1°. Meanwhile, the photocatalytic activities of the TiO_2_ nanorods were carried out using ultraviolet fluorescence spectrophotometry for the methyl orange detection, and the degradation was found to be about 71.00% ± 2.43%. Thus, TiO_2_ nanorods can be developed by a one-step acidic hydrothermal method using Ti foil simultaneously as the substrate with a TiO_2_ source; the TiO_2_ nanorods exhibited photocatalytic performance while being environment-friendly.

## 1. Introduction

Recently, many researchers have been interested in titanium dioxide (TiO_2_) because of its specific properties of high chemical stability, superior photoelectric and photocatalytic properties, medium dielectric permittivity, and low toxicity [1,2,3]. At the same time, the specific properties can be further strengthened or optimized by changing the shape morphology, phase composition, crystallite size, and surface area of TiO_2_ [4,5,6,7,8]. Thus, TiO_2_ has been intensively applied in photocatalysis, photovoltaics, solar energy conversion, sensors, textiles, paints, and cosmetics [9]. Naturally, crystallite TiO_2_ exists in three polymorphs of rutile, anatase, and brookite [10]. It has been noted that rutile TiO_2_ has better chemical stability and refractive index than the other forms [11]. Therefore, rutile TiO_2_ was chosen as the research object in this study. However, the traditional preparation processes of rutile TiO_2_ have disadvantages of toxicity and secondary pollution. To overcome these drawbacks, nano-technology for rutile TiO_2_ preparation may be a potential alternative being ecologically sustainable, renewable, and environment-friendly.

Nowadays, different forms of nano-TiO_2_ have been fabricated, such as nanorods [12], nanowires [13], nanoparticles [14], nanotubes [15], nanobelts [16], and nanoflowers [17]. Among them, nanorods have received more attention due to their chemical stability and photoelectricity [18,19,20]. Moreover, nanorods enhance the photocatalytic activity such that the recombination rate of e^−^ and h^+^ in TiO_2_ nanorods is lower than that in the other TiO_2_ structures mentioned above [21]. Many methods have been used to synthesize nano-TiO_2_, such as the template method [22], chemical vapor deposition [23], the sol-gel method [24], and the hydrothermal method [25]. Among these methods, the hydrothermal method uses a low temperature for mass production of well aligned TiO_2_ nanorods. Additionally, precise control over the phase composition and size of TiO_2_ nanorods can be obtained using the hydrothermal method [26]. Most importantly it is very convenient to produce TiO_2_ nanorods using the hydrothermal method. Patel et al. [27] used the hydrothermal method to prepare TiO_2_ nanorods with room temperature ferromagnetic (RTFM) behavior. Lv et al. prepared densely aligned TiO_2_ nanorod arrays with tunable thickness using the hydrothermal method [28]. However, some of the reported TiO_2_ nanorods were not fixed by any substrates, which could cause secondary pollution when using TiO_2_ nanorods for photocatalytic degradation. Although FTO/ITO film is often used as a substrate, TiO_2_ nanorods are easily detached from the surface of the FTO/ITO film on rinsing with water. Thus, it is necessary to use an ideal substrate to develop nano-TiO_2_ nanorods.

As a Ti source, Ti foil was considered for use as a potential substrate for growing TiO_2_ nanorods. Because of the fact that light cannot penetrate the Ti foil, the FTO/ITO glass has good light transmission, which makes researchers prefer to use FTO/ITO glass rather than Ti foil. Although the disadvantage that light cannot penetrate Ti foil is difficult to overcome, Ti foil substrate still has better properties of electrical conductivity and formability than FTO/ITO glass substrate. The Ti foil can be firmly connected to the TiO_2_ products, which makes it difficult to be washed away by water. The TiO_2_ nanorods grown directly on Ti foil have many advantages of orderly and uniform structure, low agglomeration degree, and high quantum effect. Simultaneously, obstacles of electron migration were eliminated and the light utilization of products was improved. However, to the best of our knowledge, the use of Ti foil as the surface substrate and Ti source for preparation of rutile TiO_2_ nanorods by the one-step acidic hydrothermal method has been rarely reported.

Thus, in this study, three-dimensional and dendritic rutile TiO_2_ nanorods were successfully prepared using a one-step acidic hydrothermal method on Ti foil surface, as well as a Ti source. The characterizations of the influencing factors, morphology, size, and crystal structure were also studied in detail. Furthermore, the hydrophilic and photocatalytic activities of the rutile TiO_2_ nanorods were investigated.

## 2. Materials and Methods

### 2.1. Materials

Hydrochloric acid (HCl) and nitric acid (HNO_3_) was purchased from Xilong Chemical Co. Ltd. (Shanghai, China). Ti foil was obtained from Tenghui Titanium Factory (Jinhua, China). Acetone (C_3_H_6_O) was purchased from Shanghai Yanchen Chemical Industrial Co. Ltd (Shanghai, China). Anhydrous ethanol (C_2_H_5_OH) was purchased from Tianjin Da Mao chemical reagent factory (Tianjin, China). Methyl orange (MO) was obtained from Shanghai Zhanyun Chemical Co. Ltd. (Shanghai, China). The deionized water produced using a Mili-Q Ultrapure water system with the water outlet operating at 18.2 MΩ (Millipore, Bedford, MA, USA).

### 2.2. Preparation of TiO_2_ Nanorods

The Ti foils (45 mm × 25 mm × 3 mm) were immersed in acetone solution with ultrasonic cleaning for 15 min to remove the surface oxides and residual oils. HNO_3_ (20 mL) and distilled water (30 mL) were added into a teflon-lined (100 mL) stainless steel autoclave, and placed into an electric blast drying oven at 160 °C for 4 h for removal of the impurities in the teflon-liner. After that, HCl (60 mL, 0.3–0.7 mol/L) and Ti foil were added into the teflon-lined autoclave. Ti foil was inserted vertically into the teflon-lined stainless steel autoclave and sealed. Then, stainless steel autoclave was placed in electric blast drying oven at (180–260 °C) for (8–16 h) without shaking or stirring. After the reactor cooled to room temperature, the samples were washed with distilled water and dried in a vacuum drying oven at 50 °C. Then, the samples were put into a muffle furnace and heated at 500 °C for 2 h. Finally, a green grey layer was formed on the surface of the Ti foil substrate. Meanwhile, the influence of HCl concentration (0.3, 0.4, 0.5, 0.6, and 0.7 mol/L), reaction temperature (180, 200, 220, 240, and 260 °C), and reaction time (8, 10, 12, 14 and 16 h) on the product properties were investigated.

### 2.3. Characterizations of TiO_2_ Nanorods

The morphology of the as-deposited materials was identified using an environmental scanning electron microscope (ESEM, FEI Quanta200F, FEI, Hillsboro, OR, USA). X-ray diffractometry (XRD, Bede, Durham, UK) was employed to characterize the phase structure of the samples. Transmission electron microscope (TEM) and high-resolution TEM (HRTEM) images were obtained on a JEOL-2010 HRTEM (JEOL, Tokyo, Japan) using an acceleration voltage of 200 kV. The sizes of nanorods were determined by the scale plate in the SEM and TEM images. The hydrophilic properties were observed on the optical contact angle measuring instrument (Zheke, DSA 100, Zheqi Technology, Beijing, China). The absorbance was measured by a VIS spectrophotometer (722s, Precision scientific instrument, Shanghai, China). The photocatalytic reaction was carried out in a photochemistry reaction instrument (BL-GHX-V, BILON, Shanghai, China).

### 2.4. Photocatalytic Oxidation Reactions of TiO_2_ Nanorods

The TiO_2_ nanorods sample was cut into a square with a side length of 10 mm, which was added into a quartz reaction tube with MO for the photocatalytic reaction. A 500 W high pressure mercury lamp was used as the light source. At every 15 min interval, MO solution was taken out and tested for absorbance. The degradation ratio (*η*) can be used to evaluate the photocatalytic activity with the following equation [29]:

(1) $$\eta =\frac{C_{0}-C}{C_{0}}\times 100\%=\frac{A_{0}-A}{A_{0}}\times 100\%$$

where *C*_0_ and *C* are the initial concentration and the concentration of MO solution at any time, respectively. The *A*_0_ and *A* are the initial absorbance and absorbance the of MO solution at 466 nm under UV light irradiation at any time, respectively.

## 3. Results and Discussion

### 3.1. Preparation and Photocatalytic Mechanism of TiO_2_ Nanorods

**Figure 1:**
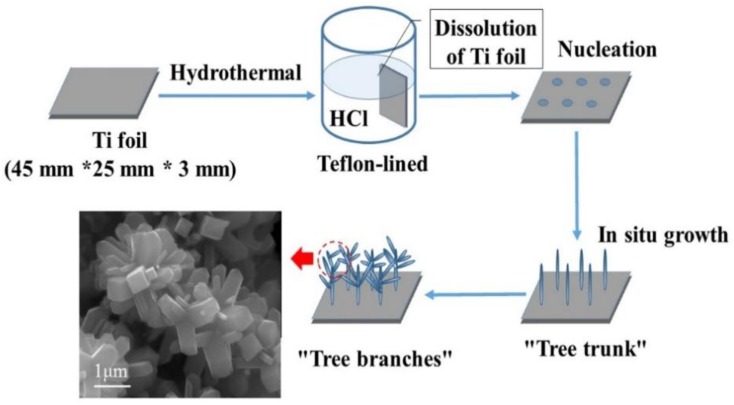
Preparation processes of TiO_2_ nanorods.

**Figure 2:**
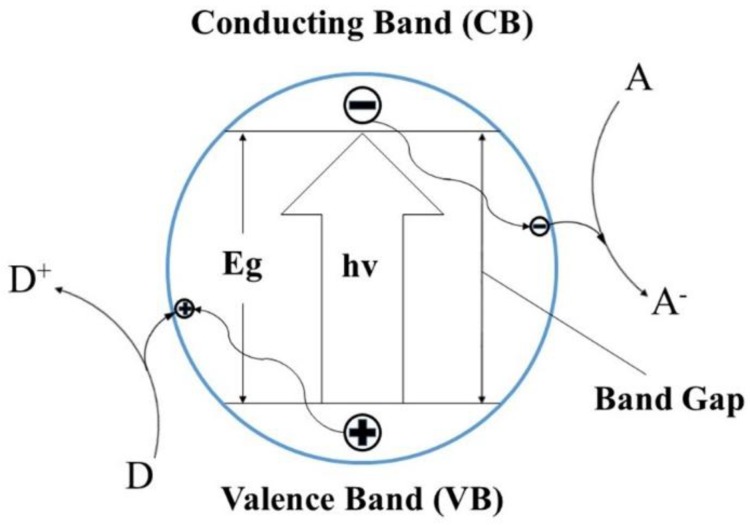
Photocatalytic mechanism of TiO_2_ nanorods.

Figure 1 and Figure 2 show the preparation processes and photocatalytic mechanism of TiO_2_ nanorods, respectively. As shown in Figure 1, Ti foil was placed into a Teflon lined autoclave and TiO_2_ nanorods grown in situ on the Ti foil surface. First, the Ti foil gradually dissolved under high temperature and pressure conditions. It can be clearly seen that several nucleations formed on the Ti foil surface. Second, a single nanorod or trunk grew out, which was the main part of the nanorod. Finally, many small branches grew on the trunk. The reason for formation was that the TiO_2_ in the solution deposited at the nucleation site to form initial nanorods, and the adsorption of the nanorods constituted the driving force for the growth of the nanorods. As the nanorods grew, the adsorption of the nanorods reached a saturated state in the longitudinal direction and diverged on the edge. Small branches were mutually constrained and eventually reached a mechanical balance. Hierarchic fractal-like branched nanorods are often obtained in acidic medium. Such branched nanorods tend to exhibit high specific surface area, which leads to better photocatalytic activity compared with ordinary rutile nanorods. Anatase mediated branching, heteronucleation, acid-assisted surface corrosion, oriented attachment, coalescence twinning, and crystal splitting are all reasons considered to be the cause of the branching in rutile according to Jordan et al. [30].

The photocatalytic degradation efficiency was attributed to the oxidative damage mainly induced by reactive oxygen species (ROS), such as (·O^2−^), H_2_O_2,_ and (·OH). These reactive oxygen species are produced on the surface of TiO_2_ when illuminated by photons with energy greater than its band gap. The electron is excited from the valance band (VB) to the conduction band (CB), and then creates an electron-hole pair (Figure 2). The holes (h^+^) react with OH^−^ and H_2_O adsorbed on the surface to form hydroxyl radicals (·OH) in the VB. The electrons (e^−^) react with O_2_ to form superoxide anions (·O^2−^) in the CB [31]. The mechanism of the radical’s generation (·OH and·O^2−^) is presented by the following equations [29]:

TiO_2_ + hv → TiO_2_ (h^+^ + e^−^) (2)

H_2_O +TiO_2_ (h^+^) →TiO_2_ + ·OH + H^+^ (3)

O_2_ +TiO_2_ (e^−^) →TiO_2_ + ·O^2−^ (4)

Therefore, the irradiated TiO_2_ photocatalysts can be used to decompose and mineralize organic compounds by the above-mentioned oxidation reactions.

### 3.2. Characterizations of TiO_2_ Nanorods

**Figure 3:**
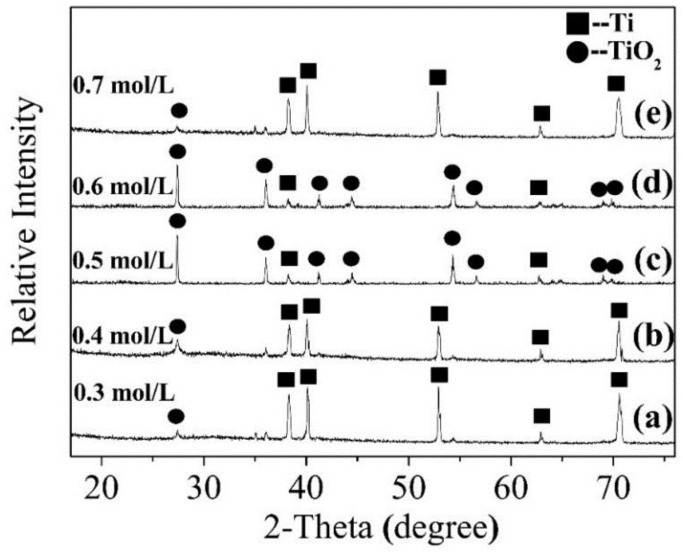
X-ray diffractometry (XRD) patterns of samples with different HCl concentration. (**a**) 0.3 mol/L; (**b**) 0.4 mol/L; (**c**) 0.5 mol/L; (**d**) 0.6 mol/L; (**e**) 0.7 mol/L.

Figure 3 shows the XRD of TiO_2_ nanorods prepared on the Ti foil surface with different HCl concentrations at 220 °C for 12 h. According to the XRD standard card of pure Ti (PDF NO. 44-1294), Figure 3 has diffraction peaks located at 38.42°, 40.17°, 53.0°, 62.94°, and 70.66°, which correspond to the (002), (101), (102), (110), and (103) planes of Ti, respectively, which indicate that the Ti substrate always exists in samples. New diffraction peaks appeared at 2θ of 36.08°, 41.24°, 44.04°, 54.32°, 56.62°, 69.0°^,^ and 69.8° in Figure 3c,d. According to the XRD standard card (PDF NO. 78-2485), the diffraction angles (2θ) of 27.43°, 36.08°, 41.24°, 44.04°, 54.32°, 56.62°, 69.0°^,^ and 69.8° correspond to the (110), (101), (111), (210), (211), (220), (301), and (112) planes for rutile TiO_2_, respectively. With increasing HCl concentration, the diffraction peaks of rutile TiO_2_ first increased and then decreased, along with the content of TiO_2_ crystal. The result shows that a rutile TiO_2_ crystal phase has formed.

**Figure 4:**
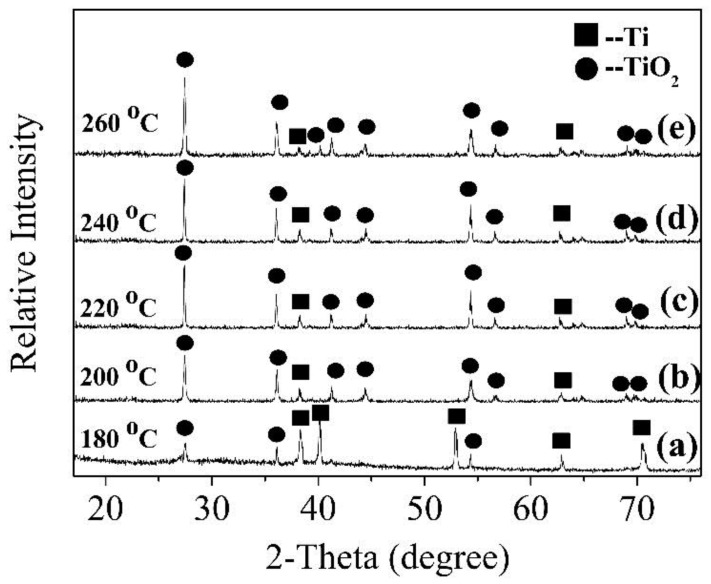
XRD patterns of samples prepared at different temperatures. (**a**) 180 °C; (**b**) 200 °C; (**c**) 220 °C; (**d**) 240 °C; (**e**) 260 °C.

Figure 4 shows the effect of the hydrothermal temperature on the development of the TiO_2_ crystal phase. These six samples were prepared under conditions of 0.5 mol/L HCl for 12 h. New diffraction peaks of rutile TiO_2_ appeared at 2θ of 41.24°, 44.04°, 54.32°, 69.0°, and 69.8° (Figure 4b). Moreover, the intensity of the main diffraction peak located at 27.43° gradually enhanced, but its growth rate was very small when the temperature exceeded 220 °C. Obviously, the intensity of the base peaks of Ti gradually weakened, and even some diffraction peaks located at 40.17°, 53.0° and 70.66° disappeared completely. It showed that with temperature increase the dissolution of Ti foil in HCl was accelerated. The increase in temperature can promote the formation of TiO_2_ until the energy provided by the temperature reaches a state of saturation for the growth promotion of TiO_2_.

**Figure 5:**
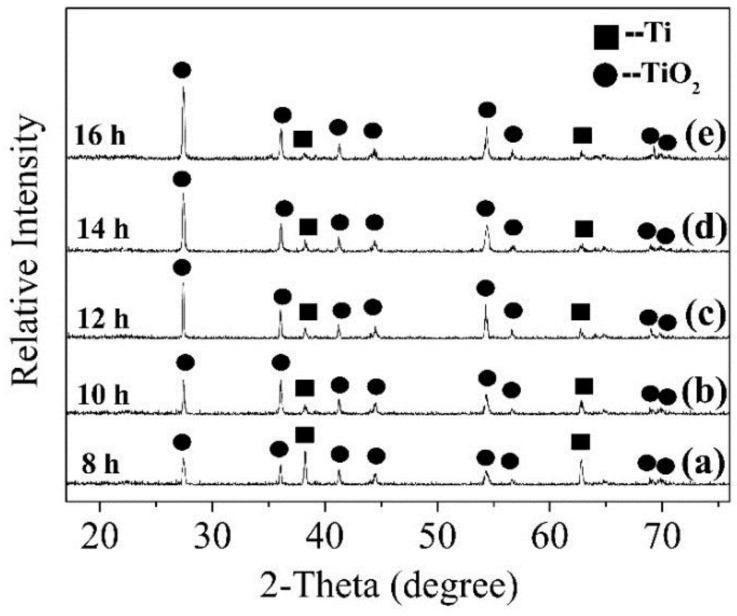
XRD patterns of samples prepared for different times. (**a**) 8 h; (**b**) 10 h; (**c**) 12 h; (**d**) 14 h; (**e**) 16 h.

Figure 5 shows the XRD of TiO_2_ nanorods prepared for different times at 220 °C with 0.5 mol/L HCl. Figure 5a shows the diffraction peaks located at 38.42° and 62.94°, which correspond to Ti according to the XRD standard card of pure Ti (PDF NO. 44-1294). However, these two diffraction peaks are significantly weakened on the reaction time increasing. On the other hand, diffraction peaks of rutile TiO_2_ appeared at 2θ of 27.43°, 36.08°, 41.24°, 44.04°, 54.32°, 56.62°, 69.0° and 69.8° (Figure 5a). Among them, the intensity of the diffraction peaks located at 27.43°, 36.08° and 54.32° is evidently reinforced. Additionally, when the reaction time exceeds 12 h (Figure 5c), the diffraction peak intensity of rutile TiO_2_ does not change significantly and finally it tends to be stable. This series of changes in the XRD pattern show that the Ti foil is consumed as the reaction time increases, but this consumption phenomenon does not last. The increased reaction time causes the Ti foil to fully react with the HCl solution, which leads to the generation of TiO_2_ crystal phase. However, the TiO_2_ crystal phase grows on the surface of the Ti foil and covers it to some degree in this process. It eventually slows down the dissolution process of the Ti foil and the growth of the TiO_2_ crystal phase.

**Figure 6:**
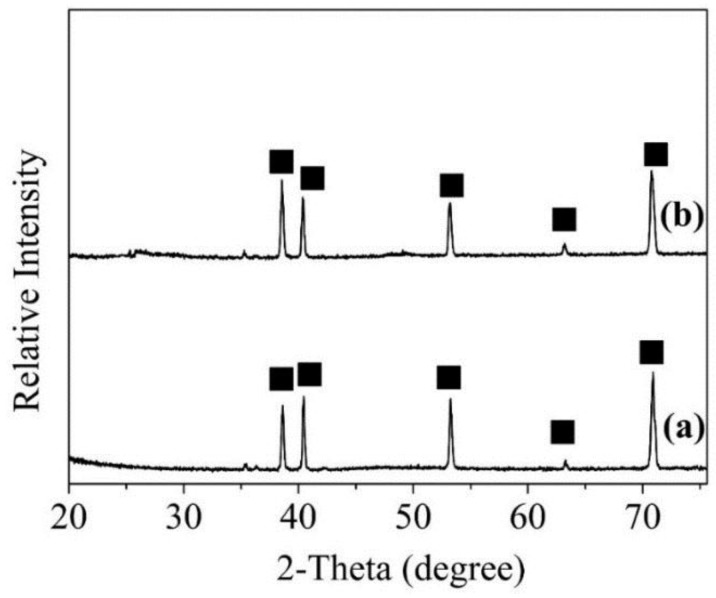
XRD patterns of Ti foil. (**a**) before reaction; (**b**) after reaction.

Figure 6 shows XRD patterns of the Ti foil before the hydrothermal reaction and after the hydrothermal reaction at 220 °C for 12 h. According to the XRD standard card of pure Ti (PDF NO. 44-1294), Figure 6a,b show diffraction peaks located at 38.42°, 40.17°, 53.0°, 62.94° and 70.66°, which correspond to the (002), (101), (102), (110), and (103) planes of Ti. Therefore, the crystal structure of Ti foil does not change significantly after the hydrothermal reaction.

**Figure 7:**
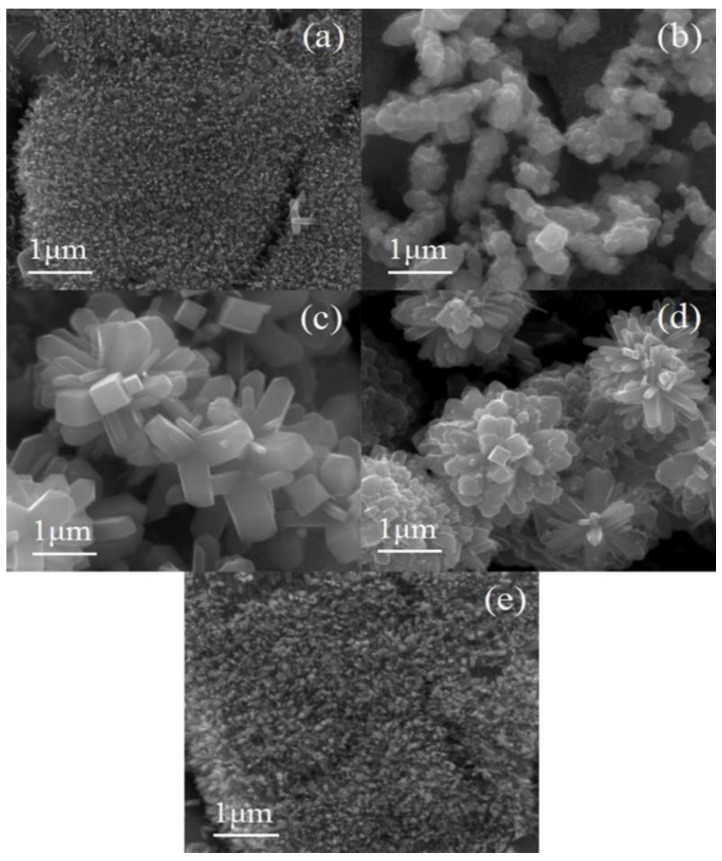
Scanning electron microscopy (SEM) images with different HCl concentration. (**a**) 0.3 mol/L; (**b**) 0.4 mol/L; (**c**) 0.5 mol/L; (**d**) 0.6 mol/L; (**e**) 0.7 mol/L.

The corresponding morphologies of the samples prepared at different HCl concentration are shown in Figure 7. As shown in Figure 7a, atoms cannot gather together to fulfill the nucleation, many small-sized particles grow on the surface of the Ti foil. Several rods with length of 100 nm were found, which indicated that it was difficult to generate when the concentration of HCl was 0.3 mol/L for the nanorods. Figure 7b,c show that nanorods have formed and many small rod-like branches have grown on the basis of the original ones with a complete rectangle. Obviously, the sample presented a three-dimensional structure, which resulted in a high specific surface area. As shown in Figure 7d, dense nanorods can be still observed, meanwhile, the length and diameter of the nanorods has become smaller. No nanorods can be found in Figure 7e when the HCl concentration increases to 0.7 mol/L. The Ti^3+^ produced by the dissolution of Ti foil gradually increases with the addition of HCl concentration. As a result, the crystalline phase content of TiO_2_ also increased and aggregated to form nanorods (Figure 7c). However, increased HCl concentration erodes the nanorods. Longitudinal nanorods were fully corroded when the HCl concentration increased to 0.7 mol/L. In this processes, the HCl concentration of the hydrothermal reaction plays an important role because the Ti foil dissolves in HCl at a hydrothermal temperature of 220 °C. Therefore, the amount of Ti^3+^ in the solution is not enough to provide the raw material for nanorod growth when the concentration of HCl is lower than 0.3 mol/L. The nucleation sites with a diameter of about 100 nm appeared (Figure 7a), but the nanorods cannot grow out significantly. The high temperature (220 °C) would provide the required energy for the growth of the nanorods, the diameter and length of the nanorods branches were about 100–500 nm and 1 μm with hydrothermal reaction conditions with increasing HCl concentration, respectively (Figure 7c). However, the aspect ratio of the nanorods at this time was about two, which means that the diameter of the nanorods was increased and would weaken the enhancement effect of the nano-branched structure on the specific surface area. In the hydrothermal reaction, the concentration of HCl was a double-edged sword for the growth of nanorods (Figure 7e). When the concentration of HCl is at 0.5 mol/L, HCl can help the growth of nanorods, but HCl can also damage the nanorods when the concentration of HCl exceeds 0.5 mol/L.

**Figure 8:**
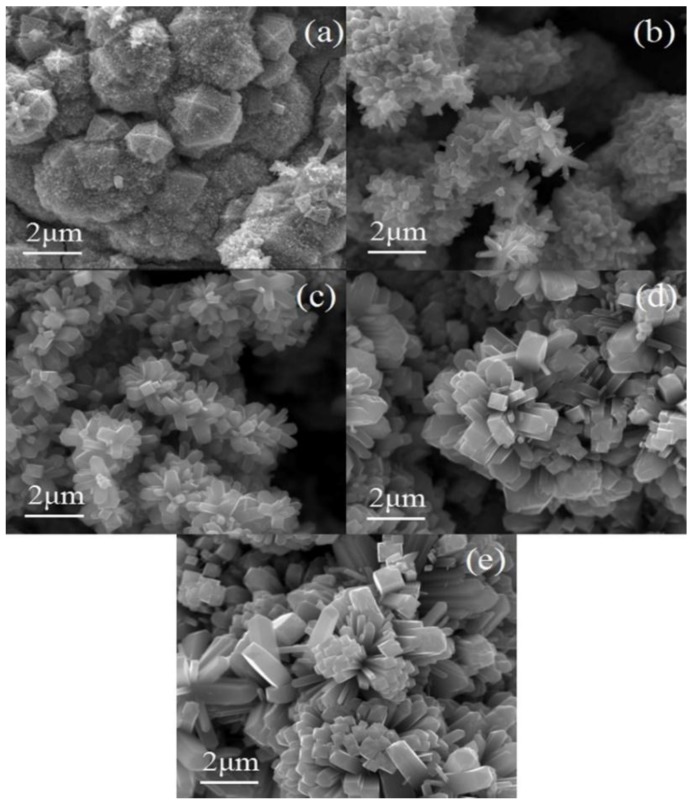
SEM images with different temperature. (**a**) 180 °C; (**b**) 200 °C; (**c**) 220 °C; (**d**) 240 °C; (**e**) 260 °C.

Figure 8a,c illustrate the progression of dysplastic nanorods developing into nanorods with intact structures in three-dimensional space. When the temperature is 180 °C, some dysplastic nanorods with diameter of 200 nm and nanosphere with diameter of about 1–2 μm grow on the surface of the Ti substrate (Figure 8a). Thereafter, the nanorods form a three-dimensional tree structure exhibiting a regular tetragonal morphology, which results in nanorods with diameter of about 100 nm and length of about 500 nm (Figure 8c). However, when the temperature continues to increase, the nanorods are tightly connected to each other when the growth of the nanorods takes place, which sharply reduces the specific surface area of the nanorods (Figure 8e,d). Obviously, the hydrothermal temperature has a direct impact on the growth of the nanorods. The low temperature cannot meet the required energy for atomic activation. Due to the insufficient energy provided, there are great obstacles for nanorod nucleation when the hydrothermal reaction temperature is at 180 °C. Therefore, a layer of TiO_2_ with an uneven surface with even nanospheres appears (Figure 8a). As the temperature increases, the atoms gain energy and the activity and migration rate significantly improve. Nanorods with diameter of 300 nm and length of about 600 nm grow on the surface of the Ti foil (Figure 8c). Subsequently, as the temperature continues to rise to 240 °C and 260 °C (Figure 8c,e), the energy provided by the hydrothermal reaction temperature causes more branched nanorods to grow and the diameter of the branched nanorods gradually increases at the same time, which results in a decrease in the aspect ratio of the branched nanorods.

**Figure 9:**
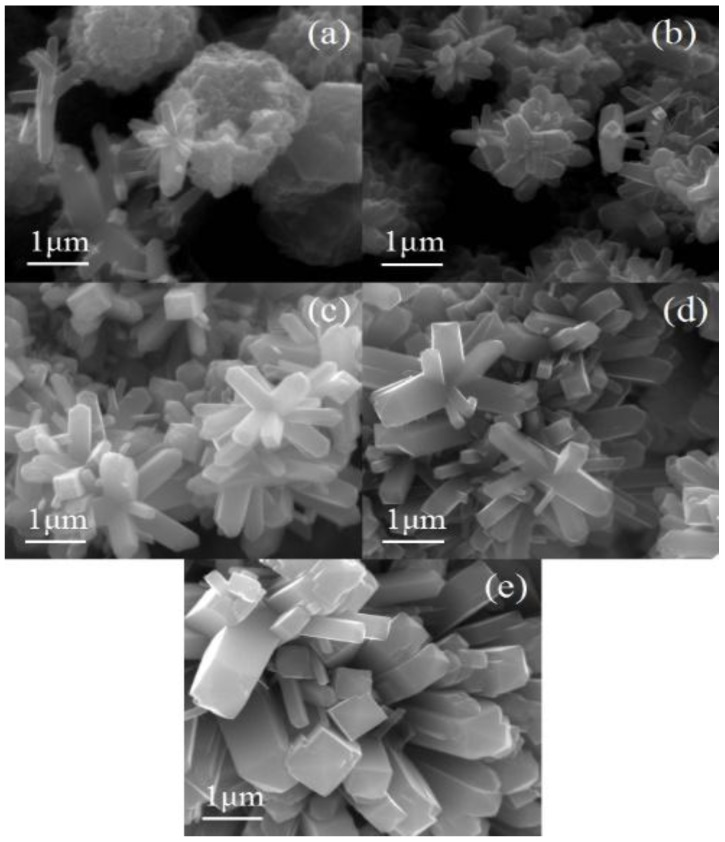
SEM images wtih different time. (**a**) 8 h; (**b**) 10 h; (**c**) 12 h; (**d**) 14 h; (**e**) 16 h.

Small amounts of non-uniform nanorods with length of 100–400 nm and diameter of 150–200 nm can be observed in Figure 9a. When the reaction time increases to 10 h (Figure 9b), many rod-like branches begin to grow in three dimensions, and the length and diameter of the branches are approximately 300–600 nm and 100–250 nm, respectively. The branched nanorods continue to grow, and the density and length are both increased, presenting a complete nanorod structure. The density and length of the branched nanorods are both increased, presenting a complete nanorod structure with length of 750 nm and diameter of 200–300 nm. With increasing reaction time (Figure 9d,e), the diameter of the nanorods becomes thicker and reaches a maximum of 1 μm. The newly grown branches are still in the initial nucleation stage, due to insufficient reaction time. Thus, the length of the nanorods is short and its structure incomplete (Figure 9a,b). During this period, both enough space for the nanorods to grow in three dimensions, and the morphology of the nanorods are best when the reaction time is 12 h. However, the space in the three directions is occupied by the continuously growing nanorods and the nanorods constrain each other in the growth direction (Figure 9d,e). Obviously, too long a reaction time does not bring beneficial effects for forming TiO_2_ nanorods. The increasing diameter results in a significant reduction in the specific surface area of the nanorods.

Thus, 0.5 mol/L, 220 °C, and 12 h were the optimum conditions for the preparation of TiO_2_ nanorods according to the results of XRD and SEM.

**Figure 10:**
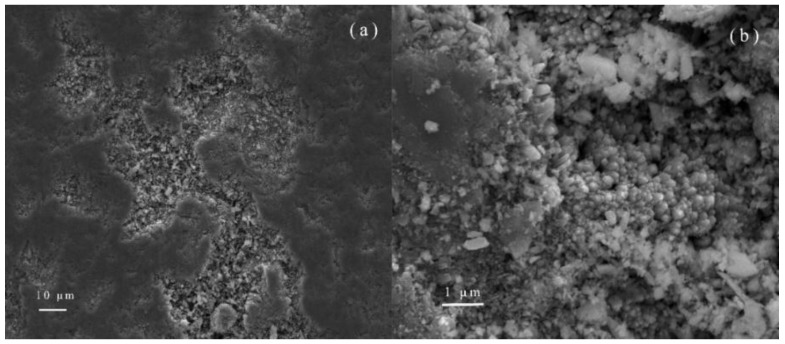
SEM images of the root section of the nanorods. (**a**) magnification 2000×; (**b**) magnification 8000×.

To convert the TiO_2_ nanorods tightly connected to the Ti foil surface, the sample was scraped off with a knife and ultrasonically cleaned for 4 h with water. The surface of the TiO_2_ nanostructure was removed and only the root portion of the nanostructure remained. The surface of the sample after the cutting process exhibited a relatively flat surface and the roots of the nanorods were exposed (Figure 10a). We can see that the root structure of the nanorods with many dense nucleation sites still exists on the Ti foil surface, due to the dissolution of the Ti foil and the aggregation of the TiO_2_ molecules. After a series of destructive treatments, the nanostructures can still be seen on the surface of the Ti foil, which proves that the TiO_2_ nanorods are tightly connected to the Ti foil in terms of mechanical properties.

**Figure 11:**
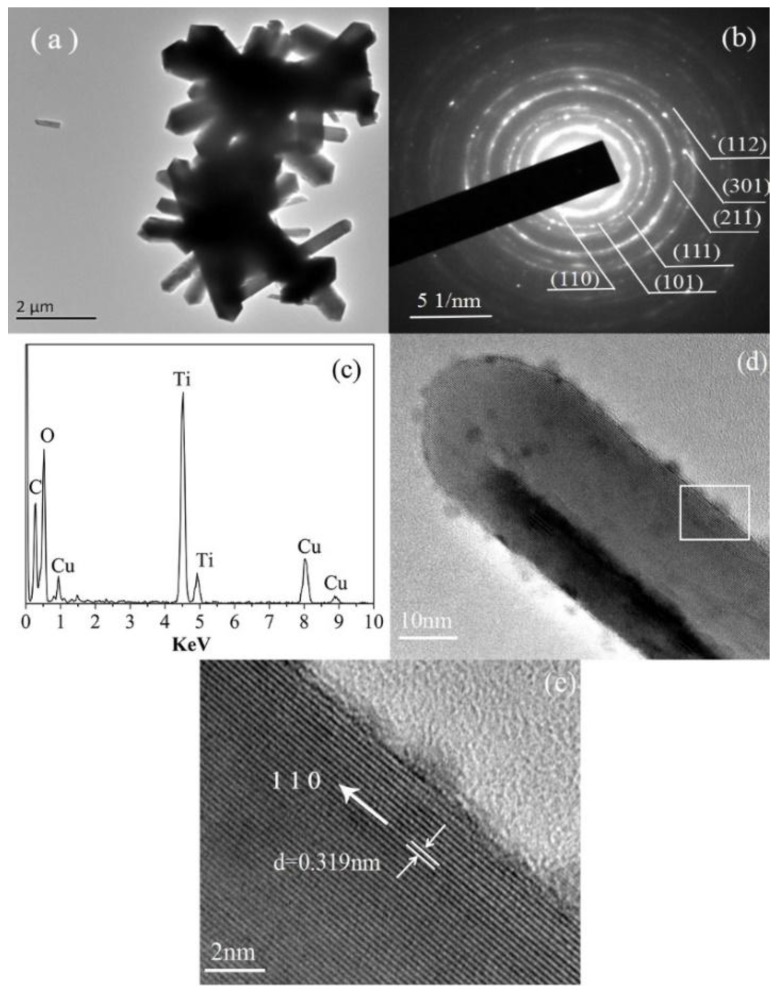
Characterization of TiO_2_ nanorods. (**a**) Transmission electron microscopy (TEM) image of TiO_2_ nanorods; (**b**) electron diffraction pattern of the TiO_2_ nanorods; (**c**) energy dispersive X-ray spectrometry (EDX) spectrum from the TiO_2_ nanorods; (**d**) TEM image of single TiO_2_ nanorod; (**e**) high-resolution TEM (HRTEM) image of the TiO_2_ nanorods.

The structures of a dendritic nanorod were studied using high TEM and the results are shown in Figure 11. It can be clearly seen that the nanorods are composed of trunks and branches (Figure 11a). The diameter of the trunk and branches is about 100–500 nm and 500 nm to 200 μm, respectively. According to the XRD standard card of TiO_2_ (PDF NO. 78-2485), the six diffraction rings were consistent with the (110), (101), (111), (211), (301), and (112) crystallographic planes of TiO_2_ (Figure 11b), respectively. Figure 11c showed the EDX spectrum of the TiO_2_ nanorods, it can be concluded that the nanorods are composed of the elements Ti and O. The Cu element came from the copper grid and the C atom from carbon film on the copper grid, respectively. A nanorod with a diameter of about 300 nm is shown in Figure 11d and it has a complete rectangular structure, which is in good agreement with the SEM images. Regular lattice fringes can be seen clearly in the HRTEM image (Figure 11e) and the *d*-spacing of the crystallographic planes is 0.319 nm, which is consistent with (110) [32] crystallographic planes of TiO_2_ (Figure 11d). The lattice fringes are parallel to the axial direction of the nanorods, indicating that the nanorods grew along the (110) plane, which is promoted by strong acid conditions and selective adsorption of Cl^−^. From these results, it can be clearly concluded that these nanorods are tetragonal rutile TiO_2_ nanorods.

### 3.3. Photocatalytic Reaction of TiO_2_ Nanorods

**Figure 12:**
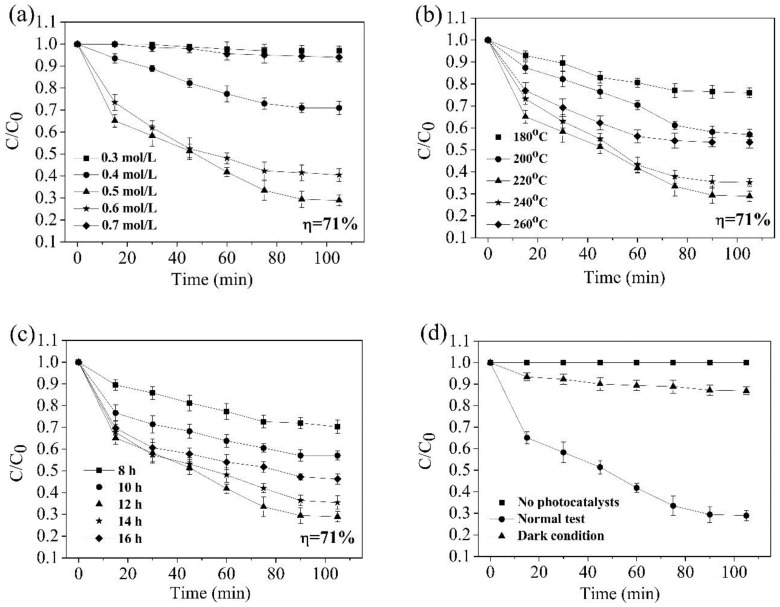
Variation of *C/C_0_* of methyl orange (MO) solution with photocatalytic time. (**a**) samples prepared at different HCl concentration; (**b**) samples prepared at different temperatures; (**c**) samples prepared for different time; (**d**) control experiment.

Figure 12 shows the photodegradation behavior of MO solution in the presence of the TiO_2_ nanorods grown at different concentrations of HCl, different temperatures, and different reaction times (Figure 12a–c). The concentration of MO is gradually reduced on increasing the HCl concentration. In other words, the degradation rate of MO solution continuously increases when the HCl concentration reaches 0.5 mol/L. MO solution can be effectively degraded with the degradation ratio reached of about 71.00% ± 2.43% after 105 min radiation (Figure 12a). However, the degradation ratio of MO decreased when the HCl concentration exceeded 0.5 mol/L, and was close to 0 when the HCl concentration of was 0.7 mol/L. High HCl concentration can corrode nanorods, which leads to a decrease in the crystalline phase content of TiO_2_ and a reduction of catalytic capacity. That is to say, the morphology of the nanorods and the content of TiO_2_ crystal phase were optimum when the concentration of HCl was 0.5 mol/L. Therefore, the degradation rate was best at this time.

Combined with XRD (Figure 4a), the crystal phase content of TiO_2_, relatively speaking, is very low, and a poor photocatalytic performance was exhibited when the temperature was 180 °C (Figure 12b). Nanorods with a complete dendritic structure can provide more active sites and enhance the scattering of incident light, and then increase the chance of contact between light and active sites when the temperature rises to 220 °C. Therefore, the photocatalytic performance of the sample was optimum at this time. TiO_2_ nanorods can be joined together to form a block with a small specific surface area when the temperature exceeds 220 °C. The gap between VB and CB widens, resulting in an increase in the degree of charge separation. The driving force required for the photocatalytic effect of the sample increases. Therefore, TiO_2_ nanorods prepared at 220 °C have the best photocatalytic performance. Similarly, the nanorods exhibited good electron transport properties, and the TiO_2_ nanorods inhibited the recombination of electron-hole pairs when the reaction time was 12 h, so the photocatalytic performance was optimal at that time (Figure 12c). Combined with SEM (Figure 9), the specific surface area and number of active sites of the nanorods are gradually reduced when the reaction time exceeds 12 h. Finally, the samples showed photocatalytic performance with a degradation rate of 71.00% ± 2.43%. At the same time, the TiO_2_ nanorods in our research study have similar photocatalytic performance compared with some previous researchers' photocatalytic studies such as D’Amato et al. [33] and Xiang et al. [34]. In contrast to Chen et al. [35], a 500 W high pressure mercury lamp could be used as one of the experimental conditions for studying photocatalytic performance. Therefore, the data from this experimental method is able to reflect the photocatalytic performance in this paper. MO solution is frequently used experimentally in many photocatalytic studies such as Shen et al. [36]. Finally, we chose methyl orange as the research reagent after careful consideration.

### 3.4. Hydrophilicity of TiO_2_ Nanorods

**Figure 13:**
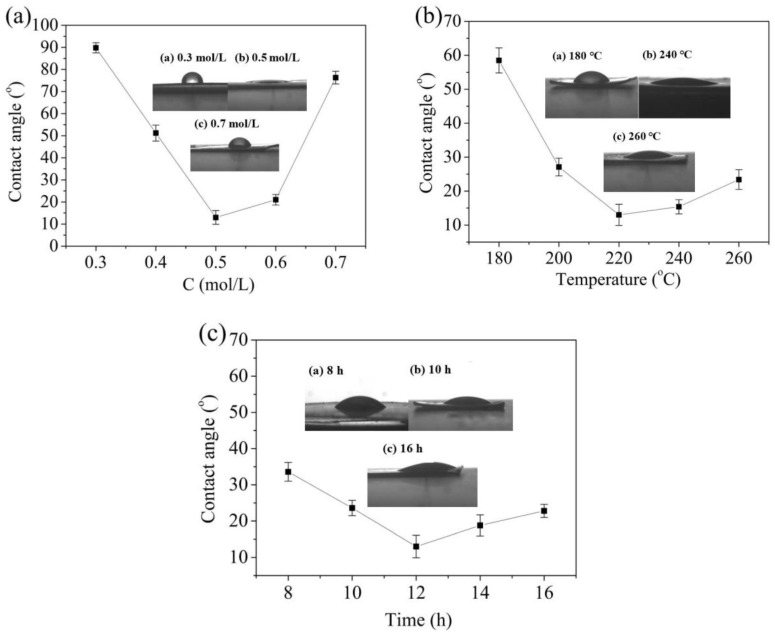
Hydrophilic property of TiO_2_ nanorods prepared at (**a**) different concentrations of HCl; (**b**) different temperatures; (**c**) different reaction times after ultraviolet irradiation.

Figure 13 shows a trend of first decreasing and then increasing contact angles. The optical contact angle of the sample was 89.8^°^ when the concentration of HCl was 0.3 mol/L. When the HCl concentration reached to 0.5 mol/L, the optical contact angle reached a minimum of 13 ± 3.1° and the water droplets were almost flat on the Ti foil. The crystalline phase content of TiO_2_ was the best and had many active sites when the HCl concentration was 0.5 mol/L, these results are in good agreement with the SEM and XRD results. Then, the optical contact angle gradually increased with the HCl concentration, which indicated that the TiO_2_ phase had been corroded by HCl. For TiO_2_ nanorods prepared at different temperature, the TiO_2_ crystal phase content was directly affected by temperature (Figure 13b). The number of electron-hole pairs was greatly reduced when the temperature exceeded 220 °C, resulting in the hydrophilic property of the sample weakening. When the temperature was 220 °C, the nanorods had the best hydrophilicity and the optical contact angle was only 13 ± 3.1°. Similarly, nanorods prepared over 12 h exhibit hydrophilicity with a contact angle of 13 ± 3.1° (Figure 13c).

The crystallinity, crystal phase content, and surface morphology of TiO_2_ have an influence on the hydrophilicity. According to the hydrophilic mechanism [37], UV-light stimulated the sample surface of TiO_2_ to produce an electron hole pair, which restored (Ti^4+^) and oxidized (O^2−^), leading to the generation of an oxygen vacancy. The vacancy reacted with surface hydroxyl groups and absorbed H_2_O, to form hydroxyl radicals. More and more H_2_O was absorbed by the hydroxyl radicals, resulting in a super-hydrophilic performance. Therefore, the hydrophilic property of samples was optimum when the HCl concentration and temperature were 0.5 mol/L and 220 °C, respectively.

## 4. Conclusions

In summary, three-dimensional dendritic rutile TiO_2_ nanorods were successfully prepared by a one-step acidic hydrothermal method using Ti foil as substrate and a Ti source. The TiO_2_ nanorods were directly grown on the conductive Ti foil and firmly held, which was beneficial for recycling without causing secondary pollution when used as a whole for photocatalytic degradation. Ti foil has a powerful induction impact on the growth of TiO_2_ nanorods. The nanorods were about 2 μm in length and diameter of about 100 nm to 1 μm. TiO_2_ nanorods grew along the [001] direction of the tetragonal rutile TiO_2_ crystal. When the HCl concentration was 0.5 mol/L, reaction temperature 220 °C, and reaction 12 h, the morphology and density of nanorods reached an optimum with hydrophilic and photocatalytic properties. The hydrophilic and photocatalytic activity of TiO_2_ nanorods reached a maximum, when the contact angle was only 13 ± 3.1° and the degradation 71.00% ± 2.43%, respectively. It can be concluded that TiO_2_ nanorods can be developed by a one-step acidic hydrothermal method using Ti foil simultaneously as the substrate and Ti source, and the TiO_2_ nanorods thus formed exhibit photocatalytic performance while being environment-friendly.
